# Supplementary material for: Splice-Junction-Based Mapping of Alternative Isoforms in the Human Proteome
Source: Cell Rep. Author manuscript; Available in PMC 2020 Jan 15. (PMC6961840; doi:10.1016/j.celrep.2019.11.026)
Supplement: 2 [file NIHMS1546469-supplement-2.zip › DF1/PXD010154/Colon/Colon_5_PPP1R12A_ISEMEEELKR.pdf]

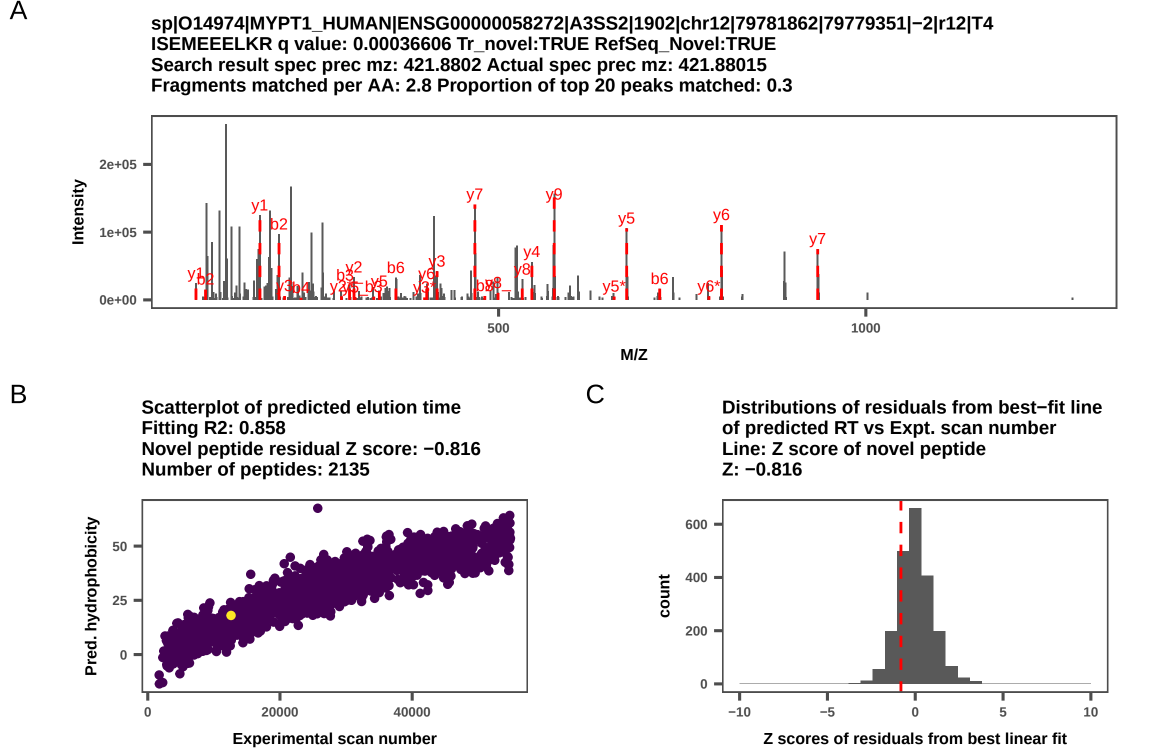
A
sp|O14974|MYPT1_HUMAN|ENSG00000058272|A3SS2|1902|chr12|79781862|79779351|−2|r12|T4
ISEMEEELKR q value: 0.00036606 Tr_novel:TRUE RefSeq_Novel:TRUE
Search result spec prec mz: 421.8802 Actual spec prec mz: 421.88015
Fragments matched per AA: 2.8 Proportion of top 20 peaks matched: 0.3
Intensity
2e+05
1e+05
0e+00
M/Z
500
1000
y1
b2
y7
y9
y5
y6
y7
y4
y8
b6
y3
y5*
y6*
B
Scatterplot of predicted elution time
Fitting R2: 0.858
Novel peptide residual Z score: −0.816
Number of peptides: 2135
Pred. hydrophobicity
50
25
0
Experimental scan number
0
20000
40000
C
Distributions of residuals from best−fit line
of predicted RT vs Expt. scan number
Line: Z score of novel peptide
Z: −0.816
count
600
400
200
0
Z scores of residuals from best linear fit
−10
−5
0
5
10
